# Digital crisis counseling during wartime: Pre–post outcomes and user experience in Ukrainian and German samples

**DOI:** 10.1016/j.invent.2026.100957

**Published:** 2026-06-01

**Authors:** Ekaterina Reymarova, Gunther Meinlschmidt, Lars Kuchinke, Melanie Eckert, Shadi Saee, Kateryna Bondar, Moritz Borrmann, Richard Wundrack, Christine Rummel-Kluge, Eva-Lotta Brakemeier, Ryan King, Anne Berghöfer

**Affiliations:** a krisenchat gGmbH, Oranienstr. 6, 10997, Berlin, Germany; b International Psychoanalytic University Berlin, Stromstr. 1, 10555, Berlin, Germany; c Department of Clinical Psychology and Psychotherapy – Methods and Approaches, Psychology, Trier University, Universitätsring 15, 54296, Trier, Germany; d Department of Psychiatry and Psychotherapy, Leipzig University Medical Center, Leipzig, Germany; e Institute of Psychology, Universität Greifswald, Franz-Mehring-Str. 47, 17489, Greifswald, Germany; f Institute of Social Medicine, Epidemiology and Health Economics, Charité – Universitätsmedizin Berlin, corporate member of Freie Universität Berlin and Humboldt-Universität zu Berlin, 10117, Berlin, Germany; g Day Clinic for Cognitive Neurology, University Hospital Leipzig, Liebigstrasse 16, 04103, Leipzig, Germany

**Keywords:** Ukraine, Digital mental health, Crisis intervention, Refugees, Psychosocial support, War trauma, Text-based counseling

## Abstract

**Background:**

Armed conflict and forced displacement are associated with elevated risks of anxiety, depression, and trauma. The ongoing war in Ukraine has exacerbated the need for mental health care while simultaneously disrupting access, underscoring the need for scalable and accessible interventions.

**Objective:**

This study examined user acceptance and changes in acute psychological distress among users of *krisenchat Ukrainian* (kcUA), a text-based digital counseling service. It explored patterns of distress levels, user satisfaction, and referral outcomes, comparing kcUA users (in Ukraine and in exile) with krisenchat Germany (kcDE) users.

**Methods:**

A pre-post observational study with a 4-week follow-up was conducted using feedback from 4436 help-seekers (2877 of kcDE and 1559 of kcUA) between July 2022 and April 2023. Surveys assessed mental health symptoms, user satisfaction, and local referral success at three time points: retrospectively assessed pre-consultation distress (“T0”), post-counseling (T1), and 4-week follow-up (T2).

**Results:**

All user groups reported significant reductions in symptoms of depression, anxiety, and stress immediately after counseling. At T2, kcUA users maintained symptom improvements, while kcDE users showed partial symptom rebound. User satisfaction and service acceptance were high across all groups (≥89% at T1). kcUA users more often reported war-related concerns and were more likely to recommend the service to others. Referral success was higher among kcDE users (46.6%) than kcUA users (34.9%).

**Conclusions:**

kcUA was associated with sustained reductions in psychological distress and was well accepted by kcUA users. Anonymous, scalable digital services may play a crucial role in supporting mental health during humanitarian crises.

## Highlights

• krisenchat Ukrainian use was associated with distress reduction in kcUA users during wartime setting.
• Symptom improvements in kcUA users were sustained after a 4-week follow-up.
• High user satisfaction (>89%) and service acceptance were observed.
• Low-threshold digital support can complement humanitarian aid.

## 1 Introduction

Armed conflicts and humanitarian crises exert profound and lasting impacts on mental health across the lifespan. Empirical studies and systematic reviews have consistently demonstrated elevated rates of anxiety, depression, and trauma-related disorders among affected populations during periods of crisis, such as pandemics and armed conflict (Charlson et al., 2019; Morina et al., 2018; Blackmore et al., 2020). For example, Charlson et al. (2019) estimated that approximately one in five people in conflict settings experience depression, anxiety disorder, post-traumatic stress disorder (PTSD), bipolar disorder, or another mental health condition.

The ongoing war in Ukraine has led to the displacement of millions, disrupting access to essential services and exposing individuals of all ages to violence and instability (UNHCR, 2024; Roberts et al., 2019). Both children and adults face heightened risks for mental health problems, with older adults, and those with pre-existing vulnerabilities being particularly susceptible (Charlson et al., 2019; Blackmore et al., 2020). These circumstances underscore the urgent need for comprehensive psychosocial support for all age groups affected by conflict and crisis.

Traditional in-person mental health services are often inaccessible during armed conflict due to infrastructural breakdown, security concerns, and population displacement (Tol et al., 2011; World Health Organization, Regional Office for Europe, 2022). Even when services are available, barriers such as stigma, language, cost, and lack of transportation can prevent affected individuals from seeking help (Naslund et al., 2017). In this context, digital mental health interventions (DMHIs) have emerged as scalable and accessible solutions. Mobile- and web-based counseling platforms can provide immediate and low-threshold support, overcoming many of the obstacles associated with traditional care (Hollis et al., 2017).

DMHIs can include self-guided psychoeducational tools, chatbot-based psychological first aid, structured brief therapies, and real-time online counseling (Mabil-Atem et al., 2024; Ng et al., 2025). Evidence from refugee and conflict-affected populations suggests that these approaches can be effective in reducing symptoms of anxiety, depression, and stress, while also supporting coping and emotional regulation (Mabil-Atem et al., 2024; Ng et al., 2025). In the context of the war in Ukraine, DMHIs have been rapidly deployed to provide scalable support, including self-help interventions targeting stress and well-being (Lahutina et al., 2024). Moreover, brief structured DMHIs have demonstrated effectiveness in reducing internalizing symptoms among displaced children and adolescents in a randomized controlled trial (Weisz et al., 2025). However, the evidence base remains limited, particularly regarding real-time, chat-based crisis counseling and its effectiveness across different populations and contexts.

One such intervention is krisenchat, a free, confidential, text-based chat counseling service for children and young adults under the age of 25, created in Germany during the COVID-19 pandemic. Krisenchat has demonstrated high feasibility and acceptability, particularly among youth without prior contact with mental health services (Eckert et al., 2022; Kohls et al., 2022). In response to the escalation of the war in Ukraine in 2022, krisenchat rapidly launched krisenchat Ukrainian (kcUA), offering culturally and linguistically tailored support primarily targeted at Ukrainians, but accessible to all people affected by the war, regardless of nationality and age. It reached individuals both within Ukraine and across the diaspora, addressing the mental health needs of those living under wartime conditions or experiencing displacement.

The present study examines the association between the use of kcUA and reported changes in acute psychological distress among displaced and non-displaced populations during wartime. Although krisenchat was developed as a low-threshold and highly confidential chat service, the study design enabled an assessment of differences in psychological distress following initial contact. By exploring reported symptom changes, user satisfaction, and referral outcomes, the study seeks to contribute to understanding how digital mental health interventions may support individuals in current and future humanitarian crises.

To address these aims, we formulated the following hypotheses:

1. Counseling concerns: Given the differing sociocultural and crisis contexts of the two services, we expected descriptive differences in presenting concerns and outcome patterns between krisenchat Germany (kcDE) users and kcUA users, reflecting differences in the circumstances of the users of all ages affected by the war vs. children and young adults living in a country without active military action. Exploratorily, we examined whether counseling concerns descriptively differed between kcUA users in exile and those not in exile.
2. Reduction in distress: Participation in chat-based counseling will be associated with a significant reduction in acute psychological distress, including symptoms of depression, anxiety, and stress, post-consultation (T1) in both: kcUA and kcDE users, as supported by evidence from digital interventions in crisis contexts (Eckert et al., 2022; Hollis et al., 2017).
3. User satisfaction: User satisfaction will be comparable to previous krisenchat samples among kcDE and kcUA users, both in and outside of Ukraine, with the majority of help-seekers (≥55%) reporting that kcUA was able to assist them well with their concerns (Eckert et al., 2022; Kohls et al., 2022).
4. Acceptance: Similar to kcDE, acceptance of the kcUA service will be comparable to previous krisenchat samples in both groups, as indicated by the majority (≥55%) of the users' willingness to recommend the service to others, consistent with previous findings on the acceptance of krisenchat (Eckert et al., 2022; Kohls et al., 2022).
5. Referral success: The success rate of referral to further healthcare among Ukrainian help-seekers already in Germany will be good (≥30%), in line with prior studies on digital referral pathways (Baldofski et al., 2023).

## 2 Materials and methods

### 2.1 krisenchat

The chat-based kcDE and kcUA services are free of charge and available around-the-clock crisis counseling services. The service allows users to remain pseudonymous, with phone numbers linked only to a masked chat ID. The service of kcDE is tailored to German-speaking children and young adults under 25. The service of kcUA is available to individuals of all ages, both within Ukraine and abroad, in Ukrainian and Russian languages via SMS and popular messaging apps such as WhatsApp and Telegram.

krisenchat's intervention consists of a chat-based crisis counseling session with a trained counselor, typically lasting approximately 60 min (Eckert et al., 2022). As a low-threshold service, it provides immediate and accessible support to individuals experiencing acute distress. The intervention focuses on emotional stabilization, co-regulation, and empathetic listening, while engaging users in collaborative problem-solving and supporting them in finding solutions (Eckert et al., 2022). Where appropriate and possible, users are referred to local or specialized support services, such as a social psychiatric service or a psychotherapist (Baldofski et al., 2023). Referral recommendations were made based on counselors' structured assessment of users' psychosocial needs, symptom severity, functional impairment, available support systems, and whether concerns exceeded the scope of brief chat-based crisis counseling, including situations involving suicidality, self-harm, trauma-related distress, or substantial psychosocial impairment. The number of sessions may vary depending on user needs and service utilization patterns, however only one consultation per 24 h is provided.

The service for both kcDE and kcUA is staffed by trained counselors, including professional and in-training psychologists, psychotherapists, and social workers. Counselors completed structured in-house training in digital crisis intervention and provided support focused on collaborative problem-solving, emotional co-regulation, and referral when necessary. The training for kcUA counselors was developed later and adapted from the kcDE training. Key differences include kcDE's integration of German emergency service procedures and its focus on youth, whereas kcUA serves individuals of all ages and does not involve emergency services. The majority of counselors at kcUA were Ukrainian nationals residing either within Ukraine or in other countries. The counselors of kcDE were located in Germany.

Participants were recruited via advertisement of krisenchat services online on social media platforms (e.g., Instagram, TikTok), as well as through distribution on the krisenchat website and partner organizations. Participants reached krisenchat via a phone number provided in these advertisements, using SMS, WhatsApp, or Telegram messaging systems.

### 2.2 Study design

This study employed a pre-post observational design with a 4-week follow-up to evaluate chat counseling provided by krisenchat, evaluating how a shared digital counseling model functioned across two distinct service settings: Ukrainian-language service (kcUA) and the German-language service (kcDE).

The evaluation of the service integrated feedback across two distinct time points with a third being a retrospective self-assessment of psychological distress before the consultation to assess both immediate and sustained intervention effects.

Counseling sessions served as each participant's starting point. Users were invited to complete a feedback survey (T1) approximately three hours after the counseling session, or the following morning if the initial session occurred overnight to ensure that the feedback survey wouldn't disrupt rest and sleep of the participants. The counseling session ended when the counselor marked the interaction as “resolved” in the system, indicating the conclusion of the specific consultation session after addressing the user's immediate concerns, providing emotional support and collaborative problem-solving, and offering an appropriate referral when necessary. Chats could also be marked as resolved following prolonged user inactivity or if the user discontinued the conversation (e.g., by blocking the service), preventing further communication.

The T1 survey included a retrospective assessment of negative affect immediately prior to counseling (“T0”), allowing for pre-post comparisons despite the absence of a baseline survey. Administration of the baseline survey before counseling could have delayed or prevented the consultation taking place.

A follow-up survey (T2) was administered four weeks after the initial counseling, capturing longer-term changes in psychological distress and overall service impact.

Ethical approval for this research was granted by the Ethics Committee of the International Psychoanalytic University on September 5th, 2022 (file reference: 2022_08).

### 2.3 Participants

The study included 4436 help-seekers who contacted either kcDE or kcUA between July 21, 2022 and April 20, 2023, and who voluntarily completed at least the first feedback survey. kcUA users were further categorized into two groups: those still in Ukraine and those living in exile (e.g., Germany, Poland, other countries). In this study, the term “in exile” is used to refer to participants residing outside of Ukraine, irrespective of their legal status (e.g., refugee status).

### 2.4 Procedure

All children, adolescents, and adults who made contact were invited to participate in the study. Prior to counseling, users received an automated information and consent message in the language of the respective service (German for kcDE; Ukrainian for kcUA) describing the privacy policy, terms of use, and voluntary participation in feedback surveys. Participation in the research surveys was entirely voluntary and required active agreement within the chat interface before users could proceed.

Participants automatically received an invitation to complete the first feedback questionnaire at least three hours after the counseling chat. This ensured that the request to participate in the study did not interfere with the counseling. If the participants agreed to be contacted again, they automatically received an invitation to feedback at T2 after 4 weeks. The survey tool for the feedback questionnaire was the open-source tool formr.org hosted at a German university. Only users who voluntarily completed the surveys were included in the analyses.

Demographic information and presenting concerns were documented by counselors during counseling sessions; all other information was obtained from participants via the surveys at multiple time points:

● “T0” assessment included at T1: A retrospective self-assessment in which participants were asked to report how they felt immediately prior to the counseling session. The measure was administered after the counseling session together with the T1 survey.
● T1 (3h - 24 h feedback): Administered approximately three hours after the counseling session, or the following morning if the initial session occurred overnight, capturing users' immediate post-session state.
● T2 (4-week follow-up): Sent via online survey links to assess the sustained impact of the counseling session over time.

#### 2.4.1 Categorization of counseling topics

Within routine krisenchat documentation, counselors assigned one or more internal topic tags following each counseling session to describe the presenting concerns discussed during the consultation. For the present study, these topic tags were grouped by the authors into seven broader thematic domains through a consensus process informed by prior krisenchat research (Eckert et al., 2022) and the operational structure of the service. These domains were used for analytical purposes and are not intended to represent formal psychiatric diagnoses:

● Mental health symptoms - Depression, self-harm, suicidality, phobia (i.e., fears related to specific situations, objects, environments, or creatures, sometimes related to panic-like symptoms)
● Psychosocial distress - Family related problems, problems with friends, problems with partner, stress, fear of future
● Emotional distress - Loneliness, lack of selfworth
● War, flight, and related migration or traumatic events - War, flight, and migration related issues
● Legal, organizational, and psychosocial support
● LGBTQIA+-related issues
● No reason specified

Counselors classified session topics and reported symptom patterns into one or more of the aforementioned internal documentation categories, consistent with the categorization approach described by Eckert et al. (2022). Counselors received internal training to ensure consistent and standardized assignment of categories, which are to be interpreted as reported symptoms, not diagnoses.

### 2.5 Material

Psychological distress was assessed using an adaptation of the STOP-D screener (Young et al., 2007), including depression (e.g., feeling sad or uninterested in life), anxiety (e.g., feeling anxious or nervous), stress, anger, and perceived lack of social support. Translation of the survey items was carried out via an expert council, where a professional translator first translated the items and research team members who were native speakers of the target language reviewed them to ensure linguistic and cultural equivalence. Each item was rated on a slider scale from 0 (not at all) to 10 (extremely). These were administered at “T0”, T1, and T2.

User satisfaction and service acceptance were assessed using the following items: “Did the chat help you?” (yes/generally yes vs. no/generally no) and “Would you recommend krisenchat to others?” (0–100 scale at T1), and “Have you already recommended krisenchat to somebody else?” (no; yes, one person; yes, multiple people at T2).

Social and living standards were assessed via an unvalidated single-item measure adapted from the MacArthur Scale of Subjective Social Status (SSS) (Adler et al., 2000); the item formulation varied slightly between kcDE and kcUA groups. The question for kcDE users was “Let's think about the education, work and money that you and your family have. How is your standard of living compared to most other people in Germany?” and for the kcUA users “Let's think about the education, work and money that you and your family had before the war. How was your standard of living compared to most other people in your home country?”

Referral success was tracked among users who received a referral recommendation during the counseling session. At follow-up (T2), users reported whether they followed the referral and whether the contact was successful. Reasons for following or not following referrals were also collected.

### 2.6 Statistical analyses

Descriptive statistics (means, SDs, and proportions) were used to summarize baseline demographic data and consultation themes for all participants, stratified by chat language (German vs. Ukrainian) and by location (in exile vs. remaining in Ukraine).

Changes in psychological distress were analyzed using paired *t*-tests between three time points: pre-counseling retrospective (“T0”), post-counseling (T1), and follow-up (T2). Adjustments for baseline values and time between measurements (in days) were performed using ANCOVAs with fixed effects. Group comparisons were conducted using ANCOVAs and linear mixed-effects models to account for repeated measures.

For user satisfaction and service acceptance, binary outcomes were created by merging “yes” and “generally yes” responses into a single “yes” category, as well as “no” and “generally no” into “no.” Differences in user satisfaction, recommendation rates, and referral success were analyzed using chi-square tests and logistic regression models. Differences in immediate service acceptance (T1: “I would recommend krisenchat”) were assessed with two-sided t-tests, while follow-up acceptance (T2: “I have recommended krisenchat”) was analyzed using chi-square tests.

All analyses were performed using R version 4.3.1 (R Core Team, 2023).

## 3 Results

### 3.1 Sample characteristics

A total of 4436 help-seekers were included into the study by completing at least the first feedback survey (“T0”, T1). Of these, 2877 were help-seekers from kcDE and 1559 participants from kcUA (including 386 in exile, 959 in Ukraine, location missing for 214). kcUA users were significantly older than kcDE users (*M* = 20.1 vs. *M* = 16.8 years, *p* < .001), congruent with the age restriction of under 25 for kcDE. Descriptively, kcUA users were more likely to be female (85.6%) compared to kcDE users (75.4%) and less likely to identify as gender diverse (1.0% vs. 4.3%). The proportion of female help-seekers was high in both Ukrainian samples, with 83.2% among those in exile and 87.6% among those residing in Ukraine. The demographic characteristics of help-seekers are presented in Table 1.

**Table 1:** Demographic characteristics of participating counseled persons (N = 4436 (100%)).

	German (*n* = 2877)	Ukrainian total (*n* = 1559)	Ukrainian living in exile (*n* = 386)	Ukrainian not living in exile (*n* = 959)
Age, mean (sd)	16.8 (3.39)	20.1 (6.89)	22.4 (8.92)	19.4 (5.75)
Median [min, max])	16.0 [8.00, 25.0]	18.0 [11.0, 61.0]	20.0 [11.0, 59.0]	18.0 [11.0, 61.0]
Not specified	10 (0.3%)	28 (1.8%)	10 (2.6%)	13 (1.4%)
Gender				
Diverse	123 (4.3%)	16 (1.0%)	8 (2.1%)	6 (0.6%)
Female	2170 (75.4%)	1334 (85.6%)	321 (83.2%)	840 (87.6%)
Male	534 (18.6%)	104 (6.7%)	30 (7.8%)	55 (5.7%)
Not specified	50 (1.7%)	105 (6.7%)	27 (7.0%)	58 (6.0%)
Chosen language for survey				
German	2852 (99.1%)	1 (0.1%)	1 (0.3%)	0 (0%)
English	22 (0.8%)	4 (0.3%)	2 (0.5%)	1 (0.1%)
Russian	2 (0.1%)	250 (16.0%)	89 (23.1%)	118 (12.3%)
Ukrainian	1 (0.0%)	1304 (83.6%)	294 (76.2%)	840 (87.6%)
Social and living standard⁎				
Worse to most other	165 (5.7%)	110 (7.1%)	40 (10.4%)	67 (7.0%)
Somewhat worse	450 (15.6%)	236 (15.1%)	68 (17.6%)	158 (16.5%)
Somewhat better	1056 (36.7%)	558 (35.8%)	155 (40.2%)	388 (40.5%)
better	516 (17.9%)	317 (20.3%)	87 (22.5%)	218 (22.7%)
Not specified	690 (24.0%)	338 (21.7%)	36 (9.3%)	128 (13.3%)

Note. M = Mean; SD = Standard deviation; Min = Minimum; Max = Maximum. Percentages are column-wise.

⁎ Values are descriptive only, no inferential comparisons performed.

### 3.2 Counseling concerns

Descriptively, counseling concerns differed between kcUA and kcDE users: kcUA users reported psychosocial distress (70.3%), emotional distress (35.9%), and war-related burdens (22.3%) more frequently, whereas kcDE users more often reported mental health symptoms (57.3%) and suicidality (19.0%) as seen in Table 2.

**Table 2:** Presenting concerns of counseled persons by group (multiple responses possible) by number and percentage of total group. Most common reasons within categories listed.

	German (n = 2877)	Ukrainian total (*n* = 1559)	Ukrainian living in exile (*n* = 386)	Ukrainian not living in exile (n = 959)
**Mental health symptoms**	1649 (57.3)	835 (53.6)	209 (54.1)	511 (53.3)
Depression	490 (17.0)	227 (14.6)	58 (15.0)	138 (14.4)
Self-harm	602 (20.9)	104 (6.7)	27 (7.0)	61 (6.4)
Suicidality	546 (19.0)	153 (9.8)	48 (12.4)	82 (8.6)
Phobia	184 (6.4)	300 (19.2)	74 (19.2)	190 (19.8)
**Psychosocial distress**	1528 (53.1)	1096 (70.3)	266 (68.9)	693 (72.3)
Family problems	560 (19.5)	363 (23.3)	101 (26.2)	221 (23.0)
Problems with friend(s)	256 (8.9)	182 (11.7)	38 (9.8)	121 (12.6)
Problems with partner	245 (8.5)	230 (14.8)	43 (11.1)	159 (16.6)
Stress	175 (6.1)	258 (16.5)	67 (17.4)	161 (16.8)
Fear of future	78 (2.7)	226 (14.5)	53 (13.7)	138 (14.4)
**Emotional distress**	808 (28.1)	559 (35.9)	134 (34.7)	341 (35.6)
Loneliness	158 (5.5)	171 (11.0)	55 (14.2)	85 (8.9)
Lack of selfworth	262 (9.1)	227 (14.6)	44 (11.4)	146 (15.2)
**War, flight, and related migration or traumatic events**	9 (0.3)	347 (22.3)	134 (34.7)	168 (17.5)
Burdened by war or refugee events	1 (0.0)	190 (12.2)	88 (22.8)	68 (7.1)
**Request for legal, organizational, or psychosocial support**	252 (8.8)	35 (2.2)	16 (4.1)	12 (1.3)
**LGBTQIA+ related problems**	110 (3.8)	57 (3.7)	14 (3.6)	35 (3.6)
**No reason specified**	129 (4.5)	30 (1.9)	8 (2.1)	18 (1.9)

Abbrev.: **LGBTQIA+**, lesbian, gay, bisexual, transgender, queer/questioning, intersex, asexual; “+” denotes additional sexual orientations and gender identities.

### 3.3 Reduction in distress

Both kcDE and kcUA users reported significant reductions in self-reported mental health symptoms following the chat-based counseling session (T1). From retrospectively assessed baseline (“T0”) to post-intervention (T1), decreases in depression, anxiety, and stress were reported in both groups, with similar magnitudes in anxiety reduction (kcDE: −1.89; kcUA: −1.87), but slightly greater improvements among kcDE users in depression (−1.92 vs. –1.38) and stress (−2.05 vs. –1.81) as illustrated in Table 3.

**Table 3:** Self-assessed severity of mental health problems of participating counseled persons (*N* = 4436) at the respective assessment points (selected STOP-D-items, mean and SD, difference between “T0” and T1, T1 and T2, *p*-value, and effect size).

	kcDE users									kcUA users								
	n = 2877	*n* = 2877	*n* = 1062							n = 1559	n = 1559	*n* = 524						
	T0	T1	T2	T1-T0	p-value	Cohen's d	T2-T1	p-value	Cohen's d	T0	T1	T2	T1-T0	p-value	Cohen's d	T2-T1	p-value	Cohen's d
Feeling sad, down, or uninterested in life	7.34 (1.90)	5.45 (2.48)	6.44 (2.47)	−1.92 (−2.01;-1.83)	<0.0001	−0.83	1.06 (0.88; 1.23)	<0.0001	0.37	6.43 (2.24)	5.07 (2.58)	4.95 (2.64)	−1.38 (−1.50; −1.26)	<0.0001	−0.60	0.02 (−0.24; 0.29)	0.8670	0.01
Feeling anxious or nervous	6.61 (2.40)	4.75 (2.79)	6.15 (2.50)	−1.89 (−1.99; −1.78)	<0.0001	−0.71	1.46 (1.26; 1.65)	<0.0001	0.47	6.82 (2.11)	4.97 (2.70)	5.48 (2.41)	−1.87 (−2.00; −1.73)	<0.0001	−0.73	0.81 (0.54; 1.08)	<0.0001	0.27
Feeling stressed	6.88 (2.40)	4.86 (2.81)	6.48 (2.39)	−2.05 (−2.15; −1.94)	<0.0001	−0.75	1.74 (1.53; 1.94)	<0.0001	0.53	6.74 (2.14)	4.94 (2.72)	5.30 (2.50)	−1.81 (−1.94; −1.68)	<0.0001	−0.72	0.56 (0.29; 0.82)	<0.0001	0.19
Feeling angry	4.35 (3.07)	3.06 (3.03)	4.88 (2.77)	−1.25 (−1.36; −1.15)	<0.0001	−0.45	2.08 (1.87; 2.29)	<0.0001	0.61	4.86 (2.79)	3.56 (2.94)	4.66 (2.62)	−1.31 (−1.45; −1.17)	<0.0001	−0.50	1.28 (0.98; 1.59)	<0.0001	0.37
Not having the social support	6.77 (2.58)	4.37 (2.92)	5.59 (2.91)	−2.45 (−2.57; −2.34)	<0.0001	−0.82	1.36 (1.14; 1.58)	<0.0001	0.39	5.95 (2.74)	4.37 (2.91)	4.75 (2.79)	−1.63 (−1.77; −1.49)	<0.0001	−0.60	0.71 (0.43; 1.00)	<0.0001	0.22
	kcUA living in Exile									kcUA not living in exile								
	n = 386	n = 386	*n* = 129							n = 959	n = 959	*n* = 370						
	T0	T1	T2	T1-T0	p-value	Cohen's d	T2-T1	p-value	Cohen's d	T0	T1	T2	T1-T0	p-value	Cohen's d	T2-T1	*p*-value	Cohen's d
Feeling sad, down, or uninterested in life	6.72 (2.16)	5.40 (2.59)	5.20 (2.55)	−1.36 (−1.56; −1.08)	<0.0001	−0.56	−0.20 (−0.70; 0.30)	0.4380	−0.07	6.34 (2.27)	4.90 (2.56)	4.84 (2.66)	−1.44 (−1.58; −1.30)	<0.0001	−0.64	0.07 (−0.24; 0.39)	0.6459	0.02
Feeling anxious or nervous	6.98 (2.15)	5.36 (2.80)	5.80 (2.41)	−1.62 (−1.86; −1.38)	<0.0001	−0.66	0.64 (0.06; 1.22)	0.0310	0.20	6.79 (2.08)	4.79 (2.65)	5.36 (2.40)	−2.01 (−2.17; −1.84)	<0.0001	−0.78	0.87 (0.55; 1.18)	<0.0001	0.29
Feeling stressed	6.95 (2.18)	5.26 (2.76)	5.48 (2.50)	−1.68 (−1.93; −1.44)	<0.0001	−0.69	0.22 (−0.34; 0.79)	0.4397	0.07	6.66 (2.12)	4.78 (2.68)	5.22 (2.49)	−1.88 (−2.04; −1.72)	<0.0001	−0.74	0.68 (0.37; 0.98)	<0.0001	0.23
Feeling angry	4.89 (2.80)	3.78 (2.97)	4.68 (2.73)	−1.11 (−1.37; −0.85)	<0.0001	−0.43	1.02 (0.38; 1.65)	0.0019	0.29	4.82 (2.78)	3.41 (2.91)	4.67 (2.59)	−1.41 (− 1.57; −1.24)	<0.0001	−0.54	1.40 (1.05; 1.76)	<0.0001	0.42
Not having the social support	5.98 (2.85)	4.52 (2.97)	5.43 (2.70)	−1.47 (−1.72; −1.21)	<0.0001	−0.58	1.22 (0.68; 1.76)	<0.0001	0.30	5.97 (2.73)	4.24 (2.89)	4.50 (2.78)	−1.73 (−1.91; −1.55)	<0.0001	−0.62	0.54 (0.20; 0.89)	<0.0001	0.17

Adjusted comparison revealed that kcUA users reported significantly higher residual symptom severity at T1 compared to kcDE users in several domains: depression (*p* = .008), stress (*p* = .034), anger (*p* = .031), and perceived lack of social support (*p* < .001).

At the four-week follow-up (T2), differing symptom trajectories were observed across the two service contexts. kcDE users showed a marked increase in symptom severity across all measured domains (e.g., stress: +1.74). In contrast, kcUA users generally maintained the initial reductions achieved at T1 (except for anger) and showed comparatively smaller increases in symptom severity between T1 and T2 across several domains. Despite these differences in symptom course, both groups benefited substantially from the intervention immediately following the counseling session.

The second part of Table 3 examines symptom reduction among kcUA users residing in exile compared to those residing within Ukraine. Significant reductions in psychological distress from retrospectively assessed baseline (“T0”) to post-intervention (T1) were observed in both groups, indicating that chat-based counseling was associated with immediate improvements irrespective of location.

Table 4 presents the results of group comparisons between kcDE users, kcUA users overall, and kcUA users by residence status (in exile vs. not in exile). Significant differences between groups are outlined in Table 4. These comparisons were conducted using ANCOVAs and linear mixed-effects models to account for repeated measures over time.

**Table 4:** Difference in change of self-assessed severity of mental health problems at T1 between kcDE and kcUA users, ANCOVA, adjusted for value at “T0” and time between “T0” and T1 (mean, 95% confidence intervals, p-value, and partial eta2).

	kcDE	kcUA	Mean difference kcDe - kcUA	*p*	partial eta2		kcUA in Exile	kcUA not in Exile	Mean difference Exile - Not in Exile	*p*	partial eta2
**Directly after counseling (T1)**	**(n** **=** **2877)**	**(n** **=** **1559)**				**Directly after counseling (T1)**	**(n** **=** **386)**	**(n** **=** **959)**			
Feeling sad, down, or uninterested in life	5.24 (5.16; 5.33)	5.44 (5.32; 5.55)	−0.19 (−0.34; −0.05)	0.0083	0.00	Feeling sad, down, or uninterested in life	5.22 (5.01; 5.44)	4.97 (4.83; 5.10)	0.26 (0.01; 0.51)	0.0456	0.01
Feeling anxious or nervous	4.79 (4.69; 4.88)	4.90 (4.77; 5.03)	−0.11 (−0.27; 0.04)	0.1575	0.00	Feeling anxious or nervous	5.27 (5.04; 5.52)	4.82 (4.67; 5.04)	0.46 (0.18; 0.75)	0.0014	0.01
Feeling stressed	4.83 (4.73; 4.92)	5.00 (4.87; 5.13)	−0.17 (−0.33; −0.01)	0.0336	0.00	Feeling stressed	5.14 (4.90; 5.37)	4.83 (4.68; 4.98)	0.30 (0.02; 0.58)	0.0334	0.00
Feeling angry	3.18 (3.08; 3.27)	3.35 (3.23; 3.48)	−0.18 (−0.34; −0.02)	0.0305	0.00	Feeling angry	3.75 (3.51; 3.99)	3.43 (3.28; 3.58)	0.32 (0.04; 0.60)	0.0235	0.00
Not having the social support	4.22 (4.12; 4.32)	4.64 (4.51; 4.78)	−0.42 (−0.59; −0.25)	<0.0001	0.01	Not having the social support	4.51 (4.27; 4.72)	4.24 (4.09; 4.40)	0.27 (−0.02; 0.56)	0.0691	0.00
**Four weeks after counseling (T2)**	**(n** **=** **1062)**	**(n** **=** **524)**			Partial eta2	**Four weeks after counseling (T2)**	**(n** **=** **1062)**	**(n** **=** **524)**			Partial eta2
Feeling sad, down, or uninterested in life	6.32 (6.17; 6.47)	5.19 (4.98; 5.41)	1.13 (0.86; 1.39)	<0.0001	0.05	Feeling sad, down, or uninterested in life	5.04 (4.60; 5.48)	4.90 (4.64; 5.16)	0.14 (−0.36; 0.65)	0.5881	0.00
Feeling anxious or nervous	6.18 (6.03; 6.33)	5.42 (5.21; 5.63)	0.76 (0.51; 1.01)	<0.0001	0.02	Feeling anxious or nervous	5.79 (5.39; 6.19)	5.36 (5.13; 5.60)	0.42 (−0.04; 0.88)	0.0736	0.01
Feeling stressed	6.48 (6.33; 6.62)	5.29 (5.09; 5.50)	1.18 (0.93; 1.43)	<0.0001	0.06	Feeling stressed	5.41 (4.99; 5.83)	5.24 (4.99; 5.49)	0.17 (−0.32; 0.65)	0.5055	0.00
Feeling angry	4.96 (4.80; 5.12)	4.48 (4.25; 4.71)	0.48 (0.20; 0.76)	0.0008	0.01	Feeling angry	4.60 (4.15; 5.04)	4.70 (4.44; 4.96)	−0.10 (−0.62; 0.42)	0.7109	0.00
Not having the social support	5.48 (5.31; 5.65)	4.94 (4.70; 5.18)	0.54 (0.25; 0.84)	0.0003	0.01	Not having the social support	5.37 (4.91; 5.82)	4.53 (4.26; 5.79)	0.83 (0.30; 1.36)	0.0021	0.02

### 3.4 User satisfaction and acceptance

At T1, user satisfaction was descriptively high in both groups. A large majority of kcUA users (91.7%) and kcDE users (89.9%) reported that the chat was helpful; this difference was not statistically significant (*p* = .078). The average recommendation rating was similar between the groups, with a mean of 87.1 out of 100 among kcUA users and 87.0 among kcDE users as illustrated in Table 5.

**Table 5:** User satisfaction of participating counseled persons (N = 3812) at assessment points T1 and T2 (persons without answer excluded).

	kcDE		kcUA total		kcUA In exile		kcUA not living in exile		p
	(*n* = 2445)	(*n* = 857)	(*n* = 1367)	(*n* = 404)	(*n* = 376)	(n = 106)	(*n* = 930)	(*n* = 278)	
	T1	T2	T1	T2	T1	T2	T1	T2	
Did the chat help you?									
No (combined “no” and “generally no”)	246 (10.1%)	114 (13.3%)	113 (8.3%)	43 (10.6%)	31 (8.2%)	16 (15.1%)	72 (7.7%)	27 (9.7%)	0.0781⁎
Yes (combined “yes” and “generally yes”)	2199 (89.9%)	743 (86.7%)	1254 (91.7%)	361 (89.4%)	345 (91.8%)	90 (84.9%)	858 (92.3%)	251 (90.3%)	
Would you recommend krisenchat…? (Scale 0 to 100)									
	(*n* = 2464)		(*n* = 1344)		(*n* = 374)		(*n* = 925)		
Mean (SD)	87.0 (19.7)		87.1 (19.5)		88.0 (17.5)		87.2 (19.9)		0.7997#
Have you recommended krisenchat…?									
		(*n* = 772)		(*n* = 380)		(*n* = 99)		(*n* = 261)	
No		265 (34.3%)		95 (25.0%)		29 (29.3%)		59 (22.6%)	
Yes, to 1 person		304 (39.4%)		141 (37.1%)		37 (37.4%)		98 (37.5%)	
Yes, to several persons		203 (26.3%)		144 (37.9%)		33 (33.3%)		104 (39.8%)	0.0001⁎
Received advice for referral									
		(*n* = 646)		(n = 129)		(*n* = 39)		(*n* = 86)	
Did not follow		268 (41.5%)		64 (49.6%)		19 (48.7%)		42 (48.8%)	
Followed, not successful		77 (11.9%)		20 (15.5%)		6 (15.4%)		14 (16.3%)	
Followed, successful		301 (46.6%)		45 (34.9%)		14 (35.9%)		30 (34.9%)	

⁎ Chi-squared test for differences in yes/no proportions between kcDE and kcUA users.

# Two sided t-test for differences between users of kcDE and kcUA.

At T2, significantly more kcUA users reported actually having recommended krisenchat to several people compared to kcDE users (37.9% vs. 26.3%, *p* < .001).

### 3.5 Referral outcomes

Among users who received a referral recommendation, 46.6% of kcDE users and 34.9% of kcUA users reported “successfully” following the referral. Approximately 49.6% of kcUA users and 41.5% of kcDE users did not follow the referral. The most frequently cited reason for not following the referral was fear that parents would find out, reported by 43.7% of kcDE users and 26.6% of kcUA users. Additional reasons among kcUA users included lack of confidence in opening up to a stranger (17.2%) and the belief that the referral would not be helpful (14.1%). Table 6 describes reasons for not following the referral.

**Table 6:** Reasons not to contact the recommended person or agency (only subgroup that received advice to make contact and did not follow, no missing data).

	German	Ukrainian total	Ukrainian living in exile	Ukrainian not living in exile
	(*n* = 268)	(n = 64)	(*n* = 19)	(*n* = 42)
Because I am scared that my parents will find out.	117 (43.7%)	17 (26.6%)	5 (26.3%)	12 (28.6%)
Because I did not feel confident enough to open up to a stranger.	77 (28.7%)	11 (17.2%)	3 (15.8%)	7 (16.7%)
Because it is my own problem/I can deal with it by myself.	64 (23.9%)	9 (14.1%)	3 (15.8%)	5 (11.9%)
Because I am scared of being put in a psychiatric unit.	61 (22.8%)	8 (12.5%)	3 (15.8%)	3 (7.1%)
Because the situation is my own fault and I do not deserve help.	60 (22.4%)	6 (9.4%)	1 (5.3%)	4 (9.5%)
Because they will not take my concerns seriously.	57 (21.3%)	9 (14.1%)	4 (21.1%)	5 (11.9%)
Because I haven't had time yet/I will contact them soon.	28 (10.4%)	16 (25.0%)	3 (15.8%)	11 (26.2%)
Because I don't need help after all/I feel better already.	25 (9.3%)	5 (7.8%)	1 (5.3%)	4 (9.5%)
Because it's too far away or it's hard for me to get there.	18 (6.7%)	9 (14.1%)	4 (21.1%)	4 (9.5%)
Because I do not believe that the referral would help me.	15 (5.6%)	9 (14.1%)	5 (26.3%)	4 (9.5%)
Other reasons	49 (18.3%)	7 (10.9%)	1 (5.3%)	6 (14.3%)

## 4 Discussion

This study evaluated the impact of kcUA, a text-based digital mental health intervention, in providing psychosocial support to individuals affected by the war in Ukraine. By comparing results from kcUA users, both those in exile and those still residing in Ukraine, to kcDE users, we aimed to assess both immediate and longer-term outcomes across key mental health domains, user satisfaction, and referral success.

Hypothesis 1 Counseling concerns.

As hypothesized, counseling concerns differed substantially between kcUA and kcDE users. kcUA users, particularly those in exile, were significantly more likely to present with topics related to psychosocial distress (70.3%), emotional distress (35.9%), and war-related burdens (22.3%). This pattern reflects the acute and ongoing impact of the war, with a particularly high proportion of Ukrainians in exile (34.7%) seeking help for war- and flight-related issues compared to help-seekers at kcDE. In contrast, kcDE users more frequently reported mental health symptoms (57.3%) and suicidality (19.0%), which may reflect different contextual stressors, and baseline mental health challenges. Additionally, since kcDE service was restricted to the users being only children and youth under 25, the counseling concerns may differ due to the developmental factors.

Hypothesis 2 Reduction in distress.

Both kcUA and kcDE users reported reductions in self-reported symptoms of depression, anxiety, and stress immediately after the chat-based counseling session (T1). These findings are consistent with previous evidence on digital interventions in youth crisis contexts (Hollis et al., 2017; Eckert et al., 2022). Notably, while both groups improved, only the Ukrainian group sustained these improvements at the 4-week follow-up (T2) generally showing a less pronounced increase in symptom severity at the 4-week follow-up compared to kcDE users. One possible explanation for the stronger rebound effect in the German sample could be related to socioeconomic differences, as previously found by Rarey et al. (2024). Rarey et al. (2024) suggested that online counseling might be somewhat less effective in helping children and adolescents from lower socioeconomic backgrounds regulate negative emotions than it is for their higher-SES peers. (Rarey et al., 2024). Thus, it is possible that rebound effects among users of kcDE are partially influenced by socioeconomic factors, whereas such mechanisms may be less applicable to kcUA users given the distinct contextual conditions associated with armed conflict.

Another explanation could be that mental health problems were cited more frequently as the reason for seeking help in Germany. However, any underlying mental disorders cannot be improved in the long term through a few chats but require longer-term therapeutic efforts. Additionally, kcUA users may have been moving from more acute war-related stress toward post-migration, adaptation, and resettlement challenges, potentially contributing to the sustained reductions in distress reported at follow-up.

Reported reductions in distress among kcUA users may reflect the supportive, culturally attuned environment of the service rather than problem resolution. Many of the help-seekers' concerns: such as survivor's guilt, military conscription fears, or separation from family - do not lend themselves to immediate solutions. Additionally, interpretation of these symptom reductions should consider baseline severity as a potential moderator. kcUA users entered the service with high levels of distress, and previous research shows that higher baseline symptom severity predicts greater absolute improvement following psychotherapy (Driessen et al., 2010). Therefore, the more sustained improvements might partly reflect regression toward the mean, however the German sample also presented with high levels of distress and did not report sustained improvements to the same extent. Thus, given the maintenance of reduced symptoms at T2, the results suggest more than just statistical artifacts and can be important in understanding the potential of easily accessible crisis interventions in the context of an armed conflict.

Hypothesis 3 User satisfaction.

User satisfaction was descriptively high in both groups (over 89%), supporting the hypothesis that most users felt that the service helped them. This level of satisfaction is in line with previous evaluations of kc DE (Eckert et al., 2022; Kohls et al., 2022), and it highlights the acceptability of the intervention format across cultural and national contexts.

Hypothesis 4 Acceptance.

User acceptance of the service was descriptively high in both kcUA and kcDE groups, with the vast majority of users expressing a strong willingness to recommend the service immediately following the intervention. These findings are consistent with previous krisenchat research (Eckert et al., 2022; Kohls et al., 2022) and support the hypothesis that acceptance levels would be comparable across samples. Notably, while willingness to recommend was similarly high in both groups at T1, differences emerged at follow-up: a significantly greater proportion of kcUA users reported having actively recommended the service to others. This divergence between stated willingness and actual recommendation behavior may point to contextual differences in service relevance and availability. In the context of war and displacement, access to psychosocial support may be more limited, positioning kcUA as a particularly salient and needed resource. Additionally, the cultural and linguistic alignment between counselors and users in kcUA may have facilitated trust and identification with the service. The high rate of peer-to-peer recommendation among kcUA users may therefore reflect both a high level of unmet need and the importance of informal support networks in crisis contexts, where word-of-mouth dissemination can play a critical role in extending the reach of digital mental health interventions.

Hypothesis 5 Referral success.

Referral success was lower among kcUA users (34.9%) than kcDE users (46.6%), indicating substantial barriers to continued mental healthcare in populations affected by the war. For Ukrainians, access to ongoing care, such as psychiatric or psychotherapeutic services, may have been constrained by several factors. Within Ukraine, shortages of referral options likely resulted from the redirection of resources toward military efforts (UNHCR, 2025). Outside Ukraine, barriers included limited availability of services in Ukrainian or Russian and location-specific constraints, which gradually improved over the years of the war but were prominent during 2022–2023, when these data were collected (UNHCR, 2025). Survey respondents cited concerns about parental discovery, low confidence in sharing personal issues, and doubts about the helpfulness of further support as reasons for not following up on referrals. This is congruent with previous reports that stigma surrounding mental health, identified as a key obstacle to seeking care (UNHCR, 2025), could have further hindered referral uptake. The anonymity and immediacy of kcUA, which allowed users to communicate via chat without providing personal information, could have helped bypass such barriers. These findings underscore structural challenges, including linguistic, cultural, and systemic mismatches between refugee populations and available services. Digital interventions like kcUA, offering support in users' native languages, may serve as crucial first-access points for individuals not yet ready or able to engage with conventional care abroad or at times when domestic interventions are not available.

### 4.1 Implications

krisenchat was initially developed as a low-threshold, digital mental health service for youth in everyday crises during the COVID-19 pandemic. Its adaptation for Ukrainian users in the context of war and displacement suggests that such models can be extended to humanitarian settings. kcUA was implemented in response to the increased demands of an unprecedented crisis and provided accessible, culturally appropriate support during a period of acute instability.

A number of core features contributed to the success of kcUA. Its confidential and low-threshold format made it especially accessible for individuals who were uncertain or hesitant about seeking help. The service's digital infrastructure allowed for rapid scalability across borders, ensuring continuity of support for users who were displaced. Moreover, the cultural and linguistic tailoring of the platform: offering counseling in Ukrainian and Russian, using familiar channels like Telegram or Whatsapp, helped build trust and ensured that the support felt relevant and attuned to users' lived experiences, available to them from a refugee camp in Germany or a bomb shelter during an air raid in Kyiv.

These results position kcUA as a potential model for future crisis interventions, including in contexts of armed conflict, natural disasters, and forced migration. Its success supports the integration of digital mental health tools into humanitarian aid strategies.

### 4.2 Limitations

While the findings are promising, several limitations must be acknowledged. First, there are substantial differences in the broader environment between kcDE and kcUA users, including exposure to war, displacement, or living in peace, as well as the primary reasons for service use (e.g., coping with COVID-19 and developmental challenges versus acute war-related crises) and cultural backgrounds (Ukrainian versus German). Comparing German youth in a peaceful environment to Ukrainians of all ages in a war or refugee context represents a major limitation. The comparison should therefore be interpreted from the perspective of service transferability rather than group similarity: these were the available populations, and the focus was on understanding whether the service model could function across different contexts. Therefore, findings should not be viewed as direct comparisons between equivalent populations and any observed group differences must therefore be interpreted cautiously and within their respective contextual conditions. Moreover, the kcUA sample included relatively few male users, particularly given the gendered impacts of the war in Ukraine, which may constrain the applicability of results to Ukrainian men.

Second, while self-reported symptom reduction is meaningful, it may be influenced by cultural response styles or social desirability. Ukrainian respondents, for example, might report improvement out of gratitude or in line with perceived expectations.

Third, the observational nature of the data limits the ability to draw causal conclusions. Although symptom reductions were reported, it is not possible to definitively attribute these improvements to the intervention itself, particularly after four weeks. Baseline differences in distress levels between groups could also confound outcome comparisons. Furthermore, the retrospective assessment of pre-consultation distress may be subject to recall bias and response-shift effects, such that reported changes may partly reflect altered symptom appraisal rather than symptom change alone (Howard et al., 1979; Sprangers and Schwartz, 1999).

Moreover, while krisenchat is conceptualized as a single-session intervention, its low-threshold and anonymous format enables users to engage with the service multiple times. Due to confidentiality and data protection procedures, the research dataset did not allow reliable identification of repeated service use at the individual level; therefore, the frequency of multiple consultations per user could not be systematically analyzed. Consequently, improvements reported at T2 may reflect not only sustained effects of an initial session but also the cumulative impact of potential additional sessions between T1 and T2. Given the constraints of the available data, it is not possible to disentangle these effects. Future studies should investigate usage patterns and their influence on outcome trajectories.

Furthermore, psychological distress was assessed using the STOP-D screener (Young et al., 2007). While this instrument has been validated in adult populations (Larsson et al., 2026; Young et al., 2015), evidence for its validity in children and adolescents remains limited. This represents a potential limitation of the present study, given the inclusion of younger participants. However, only a small proportion of the sample was younger than 11 years (0.18%), and the mean ages in the kcDE and kcUA samples were 16 and 18 years, respectively, with the Ukrainian sample including participants up to 61 years of age. Thus, the findings are likely to be most robust for older adolescents and adults, for whom the instrument is more appropriate. Future research should aim to validate the STOP-D in younger populations.

Additionally, the data only differentiates between the help-seekers of kcUA based on their location inside or outside of Ukraine, without taking into consideration those individuals who might be internally displaced within Ukraine. Future research would benefit from gathering information on the displacement status also within Ukraine.

Finally, conducting extensive research with such a highly vulnerable population in an acute crisis situation presents inherent methodological and ethical challenges. While this constitutes a limitation, the successful collection and analysis of data under these conditions also represents a notable strength of the study.

### 4.3 Future directions

Future research should aim to better understand the specific mental health needs of Ukrainian help-seekers during wartime, particularly among subgroups facing heightened vulnerability: such as displaced youth, individuals at risk of conscription, or those coping with family separation. Identifying which aspects of krisenchat's support (e.g., emotional validation, psychoeducation, or referrals) are most effective for different user profiles could guide more targeted service design. At the same time, future studies should explore longer-term outcomes beyond four weeks to assess the durability of intervention effects and consider using validated clinical assessments to enhance methodological rigor. Additionally, future research should further investigate potential differences in intervention effects between internally displaced individuals, externally displaced individuals, and individuals not in exile, particularly with regard to contextual factors such as living conditions, access to resources, and ongoing stressors that may influence symptom trajectories. Furthermore, examining the presentation of war-related trauma, anxiety, and depression symptoms within the chat-based context could yield valuable insights into the mental health impact of the war and inform the development of more tailored interventions. Future work could also explore how AI-based chatbots, hypothetically trained on existing chat data, might complement existing chat support and offer a more resource-efficient way to reach those in need. Moreover, incorporating a stronger focus on prevention could help identify early signs of distress and strengthen protective factors before crises arise.

## 5 Conclusion

This evaluation suggests that kcUA was associated with immediate reductions in psychological distress and high user-reported satisfaction and acceptance among Ukrainian help-seekers. In humanitarian settings where traditional care may be difficult to access, low-threshold digital services may provide timely and scalable psychosocial support. The findings support further investigation of digital crisis counseling models across diverse crisis and displacement contexts.

## CRediT authorship contribution statement

GM, LK, ME, SS, KB, MB, RW, CRK, and AB designed the study. RW and SS implemented and monitored data collection. SS and RK prepared the data set and did the statistical analyses. ER prepared the manuscript. ER, GM, LK, ME, SS, KB, MB, RW, CRK, EL-B, and AB discussed results and contributed to the final manuscript.

## Ethics statement

Ethical approval was granted by the Ethics Committee of the International Psychoanalytic University on July 6, 2022 (file reference: 2022_08). The participants provided informed consent to participate in this study.

## Funding

This work was supported by the 10.13039/501100003107Federal Ministry of Health (ZMI1-2521FEP001).

## Declaration of competing interest

LK, KB, MB, EL-B, and AB declare no conflicting interests. ME, SS, RW, and ER are or were paid employees of krisenchat gGmbH. CR-K received lecture honoraria from Recordati and Servier outside and independent of the submitted work. GM received funding in the context of a Horizon Europe project from the Swiss State Secretariat for Education, Research and Innovation (SERI) under contract number 22.00094. Further, GM received funding from Wings Health Inc. in the context of a proof-of-concept study, from the Swiss Heart Foundation under project no. FF21101, from the Research Foundation of the International Psychoanalytic University (IPU) Berlin under projects no. 5087 and 5217, from the Swiss National Science Foundation (SNSF) under project no. 100014_135328, and from the Hasler Foundation under project no. 23004. GM is co-founder and holds stock in Therayou AG, which is active in the field of digital and blended mental healthcare. GM receives royalties from publishing companies as author, including a book published by Springer, and an honorarium from Lundbeck for speaking at a symposium. Furthermore, GM is compensated for providing psychotherapy to patients, acting as a supervisor, serving as a self-experience facilitator (‘*Selbsterfahrungsleiter*’), and for postgraduate training of psychotherapists and supervisors.
